# Sequencing Independent Molecular Typing of Staphylococcus aureus Isolates: Approach for Infection Control and Clonal Characterization

**DOI:** 10.1128/spectrum.01817-21

**Published:** 2022-02-09

**Authors:** Kristyna Dufkova, Matej Bezdicek, Kristina Cuprova, Dagmar Pantuckova, Marketa Nykrynova, Eva Brhelova, Iva Kocmanova, Silvie Hodova, Marketa Hanslianova, Tomas Juren, Bretislav Lipovy, Jiri Mayer, Martina Lengerova

**Affiliations:** a Department of Internal Medicine - Haematology and Oncology, University Hospital Brno, Brno, Czech Republic; b Department of Internal Medicine - Haematology and Oncology, Faculty of Medicine, Masaryk University, Brno, Czech Republic; c Department of Biomedical Engineering, Brno University of Technology, Brno, Czech Republic; d Department of Clinical Microbiology and Immunology, University Hospital Brno, Brno, Czech Republic; e Department of Infection Control and Hospital Hygiene, University Hospital Brno, Brno, Czech Republic; f Department of Neonatology, University Hospital Brno, Brno, Czech Republic; g Department of Burns and Plastic Surgery, University Hospital Brno, Brno, Czech Republic; Emory University School of Medicine

**Keywords:** MLST, MRSA, MSSA, high-resolution melting, mini-MLST, spa-typing, whole-genome sequencing

## Abstract

Staphylococcus aureus is a major bacterial human pathogen that causes a wide variety of clinical manifestations. The main aim of the presented study was to determine and optimize a novel sequencing independent approach that enables molecular typing of S. aureus isolates and elucidates the transmission of emergent clones between patients. In total, 987 S. aureus isolates including both methicillin-resistant S. aureus (MRSA) and methicillin-sensitive S. aureus (MSSA) isolates were used to evaluate the novel typing approach combining high-resolution melting (HRM) analysis of multilocus sequence typing (MLST) genes (mini-MLST) and *spa* gene (spa-HRM). The novel approach’s discriminatory ability was evaluated by whole-genome sequencing (WGS). The clonal relatedness of tested isolates was set by the BURP and BURST approach using spa and MLST data, respectively. Mini-MLST classified the S. aureus isolates into 38 clusters, followed by spa-HRM classifying the isolates into 101 clusters. The WGS proved HRM-based methods to effectively differentiate between related S. aureus isolates. Visualizing evolutionary relationships among different spa-types provided by the BURP algorithm showed comparable results to MLST/mini-MLST clonal clusters. We proved that the combination of mini-MLST and spa-HRM is rapid, reproducible, and cost-efficient. In addition to high discriminatory ability, the correlation between spa evolutionary relationships and mini-MLST clustering allows the variability in population structure to be monitored.

**IMPORTANCE** Rapid and cost-effective molecular typing tools for Staphylococcus aureus epidemiological applications such as transmission tracking, source attribution and outbreak investigations are highly desirable. High-resolution melting based methods are effective alternative to those based on sequencing. Their good reproducibility and easy performance allow prospective typing of large set of isolates while reaching great discriminatory power. In this study, we established a new epidemiological approach to S. aureus typing. This scheme has the potential to greatly improve epidemiological investigations of S. aureus.

## INTRODUCTION

Staphylococcus aureus is considered a major public health problem as an opportunistic pathogen responsible for a wide variety of infections ranging from mild skin and soft tissue infections to bacteremia and invasive infections (1).

Molecular methods based on sequencing are generally applied to type S. aureus isolates, mainly multilocus sequence typing (MLST) and spa-typing. Recently, genome-wide gene by gene typing is used to analyze the whole-genome sequence (WGS) data. Core-genome multilocus sequence typing (cgMLST) (2) and whole-genome multilocus sequence typing (wgMLST) (3) are used to compare gene variations among hundreds and thousands of loci. Despite the highest level of standardization, sequencing methods are demanding on both time and staff, and high in cost. For that reason, methods including PCR followed by high-resolution melting (HRM) instead of sequencing are becoming more popular in epidemiology (4), as they are easily accessible for routine use.

HRM analysis is a rapid and cost-effective technique performed as a one step, closed tube system. Amplification is done with intercalating dye and the post-PCR analysis is based on PCR products’ controlled melting in a temperature gradient. It results in divergent melting curves that represent individual melting alleles. HRM has been used to modify MLST, known as mini-MLST or minim typing (5). Mini-MLST targets the same genes as MLST but sequencing is replaced by HRM. A combination of individual loci’s alleles enables the isolate to be assigned as a melting type (MelT). So far, mini-MLST schemes have been published not only for S. aureus (5) but also for Klebsiella pneumoniae (6), Streptococcus pyogenes (7), Enterococcus faecium (8), Campylobacter jejuni (9), and Escherichia coli (10). In S. aureus typing, HRM was similarly used to modify spa-typing (spa-HRM) (11–18). Spa-HRM typing quickly discriminates the most frequent spa-types and has already been used in local studies; however, most studies included a small amount of tested S. aureus isolates. Despite the studies proving HRM’s ability to discriminate spa-types without the need of sequencing, they also showed limitations in discriminating between highly similar melting curve shapes as there is a high variability in the *spa* gene given by repeat sequences and a different number of repeats within a *spa* gene.

To establish a sufficient S. aureus typing method with respect to laboriousness and expense, we introduce a combination of mini-MLST and spa-HRM. The proposed approach enables isolates to be prospectively studied in a tertiary care set up. Moreover, the clusters acquired by mini-MLST are highly concordant with clusters obtained by MLST and spa-typing. Regular monitoring of both methicillin-resistant S. aureus (MRSA) and methicillin-sensitive S. aureus (MSSA) at a local level could improve infection prevention and control including outbreak investigation and subsequent treatment strategies.

## RESULTS

In total, 987 S. aureus isolates were collected between June 2016 and January 2020 comprised of 369 MRSA and 618 MSSA. The collection included (MRSA/MSSA): throat swab (*n* = 342; 54/288); nasal swab (*n* = 185; 48/137); wound culture (*n* = 149; 126/23); unspecified swab (*n* = 74; 33/41); ear swab (*n* = 37; 5/32); airway mucus (*n* = 32; 18/14); blood (*n* = 24; 9/15); urine (*n* = 23; 15/8); maternal milk (*n* = 19; 2/17); anal swab (*n* = 18; 2/16); buccal swab (*n* = 15; 13/2); sputum (*n* = 13; 11/2); conjunctival swab (*n* = 12; 7/5); environmental sample (*n* = 5; 1/4); and other diverse clinical sites (*n* = 39; 25/14).

**FIG 1:**
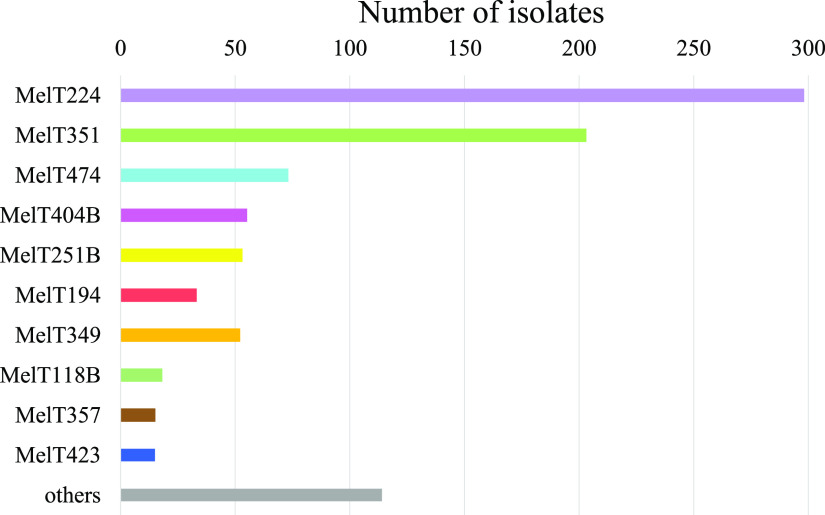
Graph indicating the population diversity of 929 S. aureus isolates in the University Hospital Brno, Czech Republic based on mini-MLST typing. The MelTs represented by less than 15 isolates are grouped together (28 MelTs).

Using mini-MLST, we were able to type 929 out of 987 isolates (94.1%). In 58 isolates (5.9%), atypical melting curves of a different shape to positive controls were repeatedly present in at least one of the mini-MLST fragments. The analysis revealed 38 MelTs in total (Fig. 1). Of the analyzed isolates, 815 (87.7%) belonged to the 10 prevalent MelTs (we defined a MelT as prevalent if the MelT appeared in 15 isolates (1.6% or more): MelT224 (*n* = 298; 32.0%), MelT351 (*n* = 203; 21.9%), MelT474 (*n* = 73; 7.9%), MelT404B (*n* = 55; 5.9%), MelT251B (*n* = 53; 5.7%), MelT349 (*n* = 52; 5.6%), MelT194 (*n* = 33; 3.6%), MelT118B (*n* = 18; 1.9%), MelT357 (*n* = 15; 1.6%), MelT423 (*n* = 15; 1.6%)). While the dominant MelT among the MRSA isolates was MelT351 (*n* = 203; 58.3%), the dominant MSSA was MelT224 (*n* = 267; 46%). The D expressing the mini-MLST discriminatory power yielded D = 0.831 (95% CI = 0.814 to 0.847).

**FIG 2:**
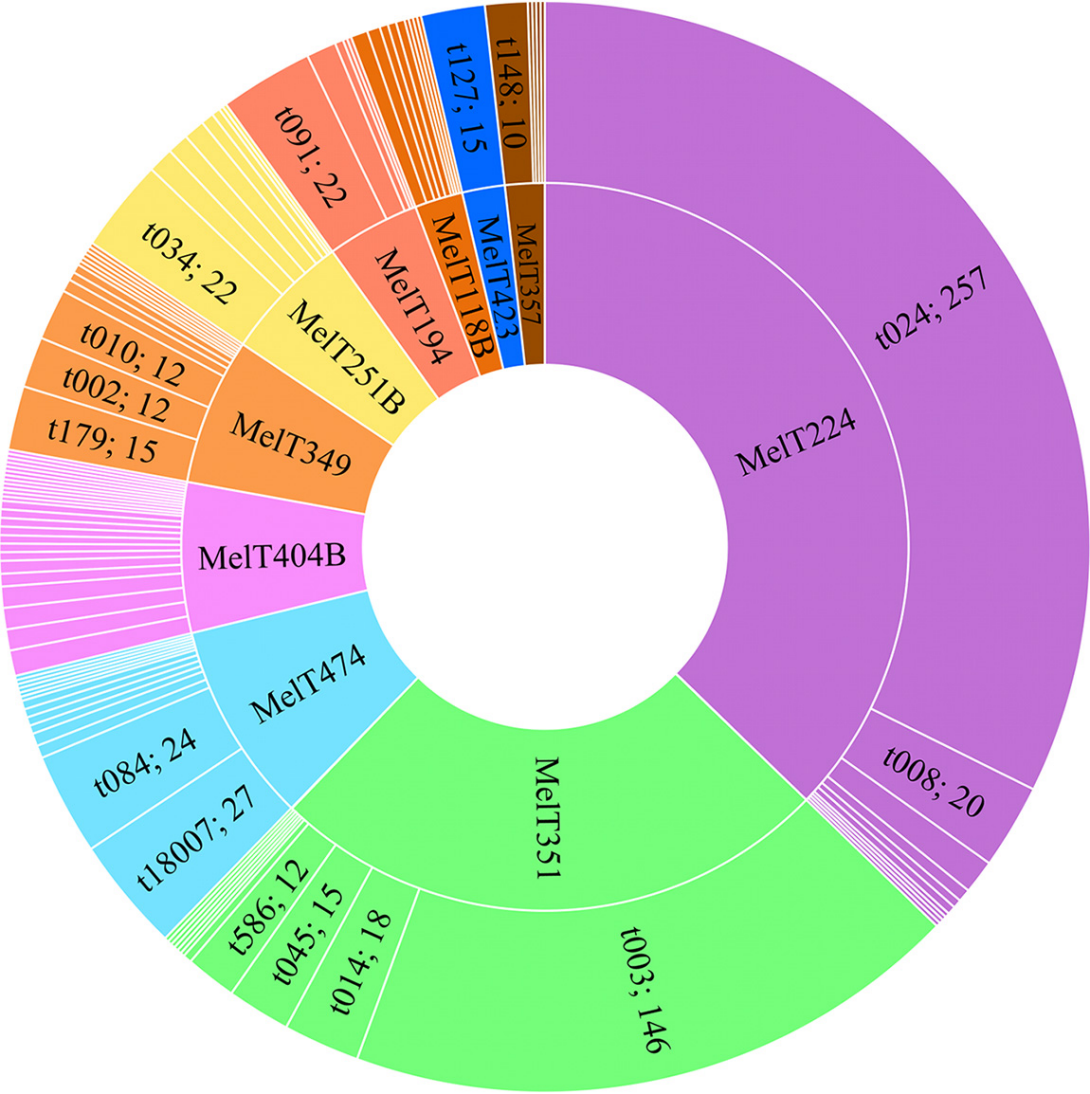
The distribution of spa-types among the most prevalent MelTs (*n* = 795; 20 isolates out of 815 were spa non-typeable). Spa-types are named when represented by more than 10 isolates. The numbers refer to the abundance of spa-types among the isolates.

To discriminate among isolates within the prevalent MelTs, spa-HRM typing was performed. In spa-HRM, we applied the modified methodology to overcome the problem with similar curves. We analyzed the spa-HRM data within the predefined MelTs separately. The spa-HRM curves with a unique shape were sequenced and a set of positive controls for each MelT was established. Subsequently, the positive controls were analyzed together with the unknown samples in each PCR run. The PCR products’ lengths were analyzed after each PCR run to reveal the number of spa-repetitions. Isolates were consequently assigned to a certain spa-type when they: (i) belonged to the same MelT; (ii) had an identical spa-HRM melting curve shape with the previously sequenced positive control; (iii) had the same PCR product length. We found out that the length analysis is crucial to differentiate among closely related or indistinguishable melting curve profiles. This method enabled 795 out of 815 isolates (97.5%) to be typed. In 20 isolates (2.5%), the spa product was not amplified and thus not further analyzed. The analysis resulted in 100 different spa-types as visualized in Fig. 2 and listed in Table S1. In our study, no spa-type was shared between different MelTs. The most prevalent MelT/spa-type combinations were: MelT224/t024 (*n* = 257; 32.3%), MelT351/t003 (*n* = 146; 18.4%), MelT474/t18007 (*n* = 27; 3.4%), MelT251B/t034 (*n* = 22; 2.8%), MelT194/t091 (*n* = 22; 2.8%), MelT224/t008 (*n* = 20; 2.5%). During the study, 10 new spa-types (MelT474/t18007, MelT404B/t18010, MelT404B/t18036, MelT351/t18037, MelT349/t18041, MelT251B/t18045, MelT349/t18046, MelT118B/t18351, MelT404B/t18694, and MelT224/t18941) and one new spa repeat (784) were revealed and reported to Ridom SpaServer. Overall, the resulting D of spa-HRM was higher than for mini-MLST alone (D = 0.831), and reached D = 0.855 (95% CI = 0.836 to 0.875).

The distribution of spa-types among MRSA and MSSA showed that the majority of MRSA belonged to MelT351/t003 (*n* = 146; 46.5%) followed by MelT251B/t034 (*n* = 22; 7.0%), MelT351/t014 (*n* = 18; 5.7%), MelT224/t008 (*n* = 15; 4.8%), and MelT351/t045 (*n* = 15; 4.8%). MSSA isolates were dominantly represented by MelT224/t024 (*n* = 254; 52.8%), followed by MelT474/t18007 (27; 5.6%), MelT194/t091 (*n* = 22; 4.6%), and MelT474/t084 (*n* = 19; 4%).

To compare the congruence between the mini-MLST and spa results, Wallace coefficient was calculated for the set of 795 isolates the results of both typing methods were available for. The results are shown in Table 1. The Wallace coefficient 1.000 indicates that two isolates with the same spa-type also shared the MelT in 100% of cases. On the contrary, the probability of two isolates that shared the same MelT also sharing the same spa-type is only 65% (Wallace's coefficient is 0.648).

**TABLE 1:** Simpson’s index of diversity and Wallace coefficient for mini-MLST and spa-HRM typing methods calculated for the 795 S. aureus isolates

Typing method	No. of types	Simpson's diversity index (95% CI)	Wallace coefficient (95% CI)	
			Mini-MLST	Spa-HRM
mini-MLST	10	0.777 (0.758 to 0.796)		0.648 (0.602 to 0.695)
spa-HRM	101	0.855 (0.836 to 0.875)	1.000 (1.000 to 1.000)	

**FIG 3:**
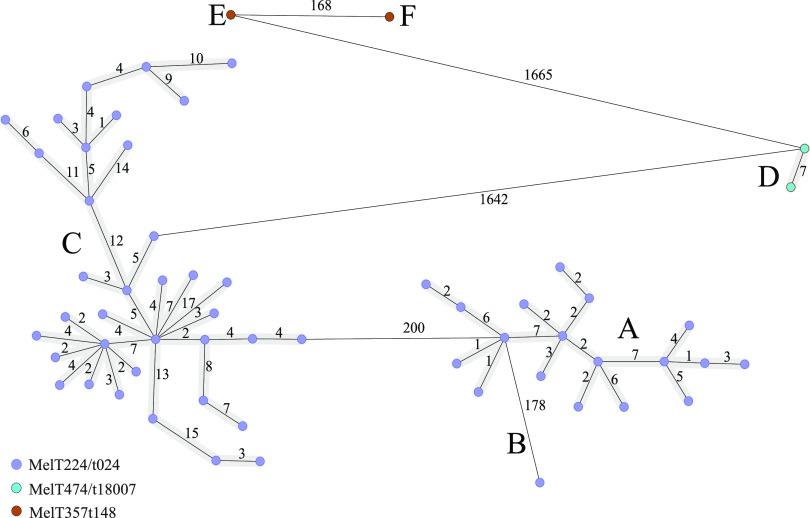
Minimum spanning tree showing cg-MLST of the 59 S. aureus isolates belonging to MelT224/t024, MelT357/t148, and MelT474/t18007. Each isolate is represented by a unique node. The lines between the circles show the number of allelic differences. The MelTs are color coded. Emergent clone MelT224/t024 is divided into two clusters, A and B, and one singleton C.

WGS was used to support the presented HRM-based methods’ ability to discriminate between closely related isolates. The set of 59 S. aureus isolates—55 representatives of cumulative detection of MSSA MelT224/t024 in premature newborns at the Department of Neonatology, and 2 + 2 randomly selected distinct clones’ representatives, MelT357/t148 and MelT474/t18007, were used for the analysis. Fig. 3 shows MST based on core- and accessory-genome allelic profiles. WGS confirmed all emergent clone isolates belong to ST8 and are closely related. However, it can be additionally divided into two clusters (A, B) and one singleton (C). The closest allele distance between cluster A (*n* = 36 isolates) and cluster B (*n* = 18 isolates) was 200 allelic differences. The internal maximum cumulative distances between the most distant isolates in cluster A and cluster B were 39 and 23 allelic differences, respectively. The singleton C differed from the adjoining cluster B in 178 targets. As evident, the HRM-based methods clearly differentiated between the emerged clone MelT224/t024 and other isolates displaying the great allelic distance between them. We individually checked the target genes obtained by WGS to detect any that might contribute to better discrimination among the observed clusters. Gene *purF* (S. aureus Accessory, SACOL1079) encoding amidophosphoribosyl transferase showed the ability to discriminate between cluster A (allele 1), cluster B (allele 398), and adjoining singleton C (allele 1141). Despite our HRM-based method being proved to have a similar level of discrimination to WGS, here we illustrate the possibility of using cg-MLST to find a marker specific for the subset of locally emerged S. aureus isolates.

**FIG 4:**
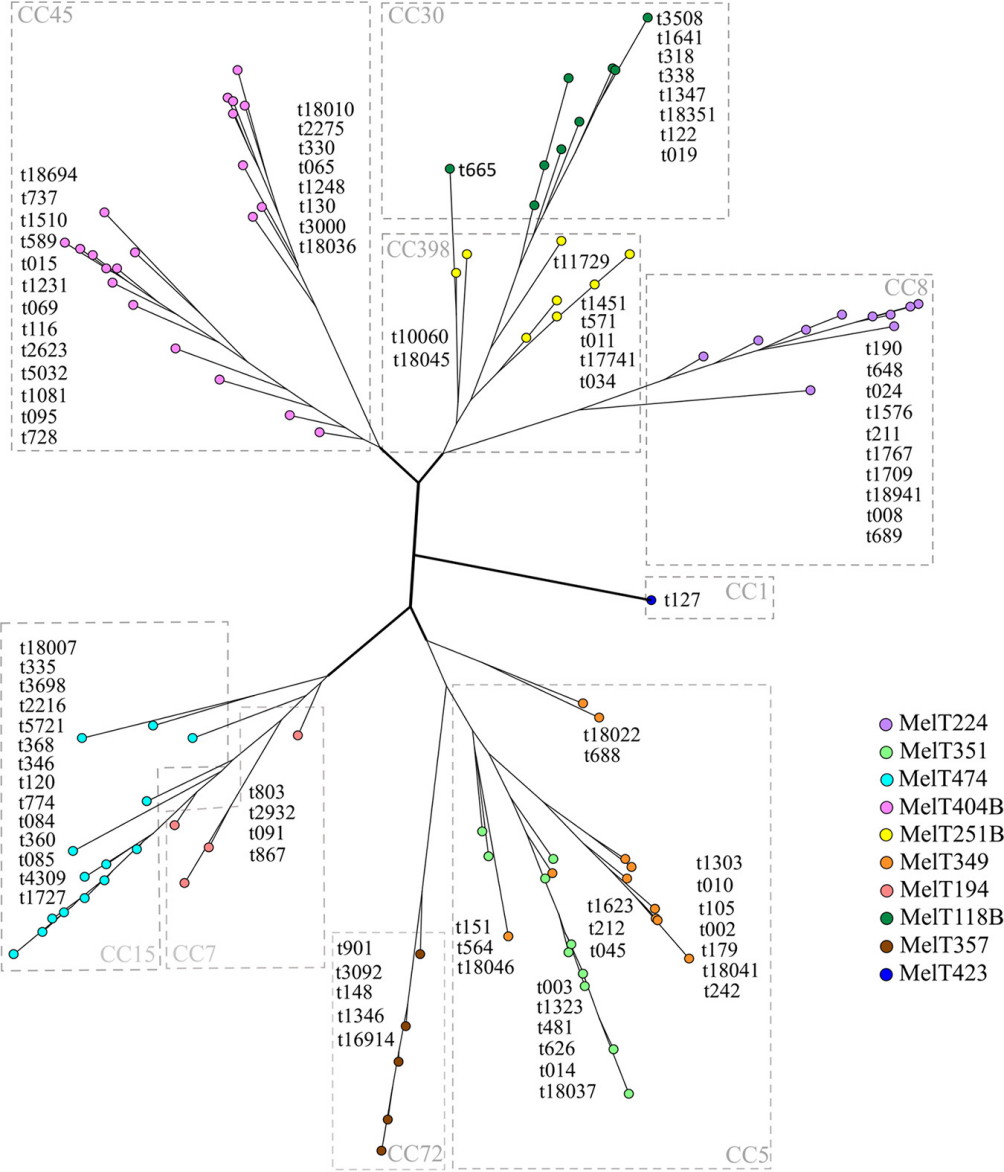
Clonality of S. aureus isolates represented by a neighbor-joining tree based on the BURP algorithm (SeqSphere+, Ridom) correlating to MLST CCs and mini-MLST. Spa-typing data from isolates belonging to the 10 prevalent MelTs (indicated by colors) were analyzed by BURP. Each spa-type is depicted with a circle and related spa-types are connected with a line. MLST CCs are indicated as DLVs in the square groups. The deeply branched parts that clearly separate distinct isolates are highlighted in bold.

To visualize the clonal structure of the S. aureus isolates, they were grouped by BURP and BURST and both compared with mini-MLST grouping. With BURP, we analyzed sequences of 94 out of 101 obtained spa-types, as they harbored five spa-repeats or more. The BURP analysis resulted in the neighbor-joining (NJ) tree showed in Fig. 4. The NJ tree contains five deep branches clearly separated from the others. The relationships among the spa-types within each branch was further analyzed by the BURST algorithm. The BURST algorithm clustered the spa-types into nine MLST-CCs: CC8, CC5, CC15, CC45, CC398, CC7, CC30, CC1, and CC72. The MLST-CCs mapped the spa relationships, as described before (19). Besides, MLST-CCs indicated excellent concordance with Melt distribution since the mini-MLST method is derived from MLST.

## DISCUSSION

In this study, we have proposed an optimized HRM-based approach for prospective S. aureus molecular typing. While the focus is primarily on resistant strains such as MRSA, vancomycin resistant S. aureus (VRSA), or linezolid resistant S. aureus (LRSA), there is generally less attention focused on MSSA worldwide. Despite the poorer responses to antibiotic therapy in MRSA infections, MSSA significantly contributes to high percentages in mortality, especially in neonates with low birth weight (20). Thus, strategies targeting both MRSA and MSSA should be applied in hospital screening and infection prevention control (21).

Contrary to sequencing-based techniques which are far from routinely used in local hospital laboratories, mainly due to laboriousness and time and cost demands, methods based on HRM analysis have several advantages (4). The proposed approach combines two HRM-based methods (mini-MLST and spa-HRM) tested on a large set of isolates and supported by WGS.

We successfully adopted a previously published mini-MLST with slight modifications. Initially, the limiting factor for using the adopted mini-MLST scheme was the absence of an updated conversion key enabling mini-MLST data to be easily linked with MLST data. To overcome this issue, we tried to update the previously published conversion key, but mainly due to errors found, we decided to construct a completely new algorithm that enables regular updates and takes account of newly described sequence types (STs) (22). Similar to the original study (5), we also obtained curves that were not predicted *in-silico*. Sequences of aroE88/155 fragment with the same G+C content thus melting in two domains were indicated as so-called half alleles. MelTs with half alleles in an aroE88/155 fragment are indicated as A or B for the presence of allele 23.5 (23 G+C) or 24.5 (24G+C), respectively. With this modification, our conversion key can also be easily used when users decide not to take half alleles into consideration. Moreover, in locus *tpi36*, we were able to distinguish between alleles 64 and 65, previously evaluated as an undistinguishable 64/65 cluster (5).

To increase discriminatory power, we successfully combined mini-MLST with a previously published spa-HRM technique (12). We confirmed that spa-HRM cannot be used as the only analysis tool. First, when a larger population of strains is analyzed, the amount of different, but extremely similar, melting curves is too high to allow trustworthy discrimination. Second, we found strains belonging to a different spa-type which produced indistinguishable melting curves. That agrees with previous studies’ results, which also point out that not all spa-types can be differentiated by HRM (11) because there are thousands of spa-types described in Ridom SpaServer (currently 20.038 different spa-types, as of July 15, 2021). Mayerhofer et al. (18) applied spiking with other spa-types’ genomic DNA, however, spiking did not lead to improved curve resolution in our case (data not shown). For that reason, analysis within a predefined MelT and analyzing the PCR product length might be necessary to discriminate between the spa-HRM curve profiles.

Altogether, the D calculated for mini-MLST was D = 0.831, and reached D = 0.856 together with spa-HRM. Combining mini-MLST and spa-HRM was only slightly higher than mini-MLST alone due to the invariability in our set of isolates caused by the clonal character.

The obtained results are expressed as MelT/spa-type; however, they can be easily linked to ST either by our conversion key or by the number of studies reporting an association of certain spa-types with STs. Our results showed a great proportion of MelT351/t003 (*n* = 146; 46.5%) among MRSA isolates, followed by MelT251B/t034 (*n* = 22; 7%), MelT351/t014 (*n* = 18; 5.7%), MelT224/t008 (*n* = 15; 4.8%), and MelT351/t045 (*n* = 15; 4.8%). The results correspond with a recent study that reported MRSA spa-type t003 dominance (30.84%) in hospitals throughout the Czech republic (23). On the other hand, they reported a lower occurrence of t034 (1.4%) and t045 (1.6%) and a considerably higher occurrence of t586 (20.7%) that we rarely reported (*n* = 12; 3.8%). Spa-type t034 has been described as the most commonly occurring spa-type identified in livestock in the Czech Republic (24), as well as reported from other European countries (25). Spa-type t045 is known for its emergence and following dissemination in Germany since 2005 (26).

MSSA isolates showed the dominance of MelT224/t024 (*n* = 254; 52.8%), followed by the newly discovered MelT474/t18007 (*n* = 27; 5.6%), further followed by MelT194/t091 (*n* = 22; 4.6%) and MelT474/t084 (*n* = 19; 4.0%). Increased detection of MelT224/t024 has not been described before. The cumulative detection of this clone among MSSA in the Department of Neonatology calls for further investigation. The occurrence of MelT194/t091 and MelT474/t084 correspond to the top MSSA spa-types described in the large European S. aureus bloodstream infection survey (27).

In our study, we were not able to use spa-typing in 20 isolates (2.5%) due to possible genetic rearrangement in the primer annealing site. This proportion relates to that previously published by Votintseva et al. (28). Along with that study, our data showed the frequently occurring spa nontypeable MelT251B (*n* = 8), analogous to ST398. This ST is common among livestock and there are frequent deletions in the IgG-binding part of the *spa* gene described.

Cumulative MelT224/t024 detection in the Department of Neonatology in recent years suggests that it is a local stable clone, well adapted to environmental survival. The combination of mini-MLST with subsequent spa-HRM revealed population structure at a sufficient level. Further WGS analysis of emerged clones distinguished between two distinct clusters and one singleton. The results suggest the use of an additional marker to fully differentiate among the emerged isolates. Gene *purF* displayed an *in-silico* ability to be selected as an additional marker, however, this has not been further analyzed in our study.

Clustering algorithms are useful for studying the isolate relatedness and describing the S. aureus population’s clonal structure (19). As for the proposed approach, mini-MLST implementation prior to spa-HRM enables not only better resolution between similar spa curve shapes, it also provides clonal structure information, as proven by comparing with the BURP and BURST results. Within each MelT, spa-types consist of highly similar repeat profiles (Table S1) explained by the loss or gain of repeats, possibly caused by slipped-strand mispairing during DNA replication (29), and rarely occurring point mutations within repeats (30). Therefore, spa repeats are likely to reflect the clonal nature of S. aureus, providing evidence about isolate relatedness and are useful in studying local evolution.

Although WGS costs are decreasing, its implementation as a routine surveillance method is time-consuming and inconvenient for clinical application. In the last decade, Sanger sequencing has become more affordable and available due to commercial providers, among other reasons. Because local hospital laboratories handle dozens of routine clinical samples a day, incorporating additional on-site typing methods calls for a reasonable approach. Despite hardly achieving a perfect resolution by non-sequencing methods, the presented combination of two HRM-based methods provided sufficient resolution of related and nonrelated strains. Those methods are single tube, so they do not require further manipulation with the sample; therefore, minimizing workplace contamination and hands-on-time. The mini-MLST enables inter-laboratory comparison because the previously sequenced positive controls with assigned allele numbers according to the G+C content are present in each run. Furthermore, such an approach helps to identify potential outbreaks by implementing WGS that would otherwise not be possible without presorting isolates. In case of emergent clones detected by HRM-based methods, WGS can provide a detailed insight into population diversity and find a marker specific for the certain local epidemiological situation. Further implementation of this additional marker together with mini-MLST and spa-HRM would enable a detailed non-sequencing analysis of the population structure in our hospital. It is necessary to keep in mind the caveats of the approach, such as lower resolution than sequencing methods and the importance to validate the HRM method carefully prior to large-scale testing.

## MATERIALS AND METHODS

This study was conducted at the University Hospital Brno, Czech Republic, a tertiary care hospital with more than 2,000 beds and 5,000 employees. There are more than 1,000,000 people treated in outpatient clinics and over 70,000 patients hospitalized every year.

### Isolates collection.

S. aureus isolates were obtained as part of prospective observational study and were analyzed anonymously. All MRSA isolates were collected throughout the hospital between June 2016 and September 2019. MSSA isolates originated from the neonates and pediatric intensive care unit patients hospitalized in the period of June 2016 to January 2020. S. aureus identification was confirmed by MALDI-TOF-MS BioTyper (Bruker Daltonics, Germany). Using the disc diffusion method, strains were identified as MRSA while resistant to oxacillin or MSSA for oxacillin sensitive strains. The pure culture colonies were suspended in sterile water and stored at −20°C for further analyses.

### DNA isolation.

The genomic DNA extraction method was different depending on the DNA quality demand. For HRM-based methods (mini-MLST, spa-HRM) and Sanger sequencing-based methods (MLST, spa-typing) the DNA was isolated using the previously published Chelex 100 Resin protocol (22). For WGS, the high-quality DNA was extracted using GenElute Bacterial Genomic DNA kit (Sigma–Aldrich, USA) according to the manufacturer’s instructions. After DNA extraction, DNA purity and concentration were measured on a NanoDrop (Thermo Fisher Scientific, USA) and Qubit (Thermo Fisher Scientific, USA), respectively.

### HRM-based molecular typing.

The proposed HRM-based methods were performed for the whole set of S. aureus isolates. Mini-MLST was performed as described by Lilliebridge et al. (5) using 2 × SensiFAST HRM mix (Bioline, UK). The thermal cycling parameters were: 95°C (3 min); 40 cycles of 95°C (5 s), 56°C (10 s), 72°C (20 s), 95°C (2 min), 50°C (20 s), followed by HRM ramping from 65 to 85°C, increasing by 0.1°C at each step. For the analyses, a Bio-Rad CFX96 Real-Time PCR Detection System (Bio-Rad, USA) or a RotorGene 6000 device (Corbett Research, Australia) were used, achieving comparable results. In each run, previously sequenced positive controls were analyzed in parallel. Acquired unique HRM curves were sequenced, the allele numbers were assigned to the associated melting allele according to the G+C content and were allocated to the positive controls with the same melting profile (Fig. S1 and Fig. S2). The allele numbers were combined to get the final MelT. Individual MelTs were named according to the novel conversion key available on our (http://www.cmbgt.cz/mini-mlst/t6353/) and is regularly updated (last update April 6, 2021) according to the Bacterial Isolate Genome Sequence Database (BIGSdb) updates (https://pubmlst.org/organisms/*staphylococcus*-aureus). The complete list of alleles predicted on the basis of G+C content and the melting curves obtained in this study are presented in Table 2.

**TABLE 2:** Mini-MLST alleles predicted from the MLST database (by April 6, 2021, *n* = 6,745 STs)^*a*^

Mini-MLST locus	Predicted mini-MLST alleles and no. of associated STs
*arcC78/210*	45 (1), 47 (1), 48 (1), 49 (1), 50 (40), **51** (1342), **52** (2268), **53** (2434), **54** (514), 55 (141), 56 (1), 76 (1)
*aroE88/155*	**21** (39), **22** (458), **23** (2404), **23.5** (227), **24** (1361), **24.5** (1808), **25** (441), 26 (7)
*gmk286*	10 (1), **11** (400), **12** (4369), **13** (1967), 14 (8)
*pta294*	38 (2), 39 (7), 40 (82), 41 (62), **42** (57), **43** (2772), **44** (2972), **45** (787), 46 (4)
*tpi36*	59 (3), 61 (35), **62** (196), **63** (238), **64** (1529), **65** (2907), **66** (1686), **67** (132), 68 (5), 69 (14)
*tpi241/243*	**42** (79), **43** (3382), **44** (3060), **45** (177), 46 (7), 47 (5), 49 (1), 50 (8), 51 (9), 52 (14), 54 (3)

a The number of associated sequence types (STs) are shown in parentheses. The alleles highlighted in bold were observed in this study.

Spa-HRM typing was made as described by Mazi et al. (12). HRM analysis of the amplicons was performed between 78°C and 87°C with a stepwise increase of 0.2°C. Acquired HRM curves were interpreted for each predefined MelT separately and compared with the previously sequenced positive controls analyzed in the same PCR run (Fig. S3 and Fig. S4). The PCR product length analysis was an additional method for spa-HRM assignment and was performed using QIAxcel (Qiagen, Germany).

### Statistical analysis.

To measure the discriminatory ability in our isolate’s collection, the Simpson’s index of diversity (D) was defined. To measure concordance between the typing methods, Wallace coefficient was used. These calculations were performed on the Comparing Partitions website (www.comparingpartitions.info).

### Sanger sequencing-based molecular typing.

The sequencing-based methods were applied to verify the positive controls and acquire sequencing data for clonal analyses. MLST was performed as previously described by Enright et al. (31). PCR products were purified using ExoSAP-IT (Thermo Fisher Scientific, USA) and sequenced using BigDye Terminator v1.1 (Thermo Fisher Scientific, USA) with an ABI 3130 Genetic Analyser (Thermo Fisher Scientific, USA) and Sequencing Analysis v5.4 software. The MLST alleles and final sequence types (STs) were assigned using the PubMLST website (https://pubmlst.org/organisms/*staphylococcus*-aureus).

Spa-typing was performed as an amplification of the protein A gene (spa) polymorphic X-region using 1095F and 1517R primers as described previously (32). Sequences were assigned to spa-types using the online spaTyper database (http://spatyper.fortinbras.us/) as well as Ridom SpaServer (https://spaserver.ridom.de/).

### Whole-genome sequencing.

The WGS library was prepared using KAPA HyperPrep Kits (Roche, Switzerland). The Illumina MiSeq platform was used for WGS and 250-bp paired-end sequencing was performed. The obtained reads were quality checked using FastQC (Babraham Bioinformatics, UK) and assembled using Burrows-Wheeler Aligner (33). Ridom SeqSphere+ (Ridom, Germany) S. aureus seed genome strain COL (NC_002951.2) was used as the reference genome. To remove unmapped reads, reads with poor quality, and duplicates, SAMtools were used (34). After reference mapping, all positions with less than 10× coverage and all ambiguous positions (less common base represented at least 10% of bases in the target position) were removed from further analysis. UGENE software was used to obtain consensus sequences (35). With SeqSphere+ (Ridom, Germany), the genomes were compared with a gene-by-gene approach using an incorporated S. aureus cgMLST scheme and S. aureus accessory genome MLST scheme comprising 1,862 core-genome genes, and 706 accessory genes, respectively (2). A minimum spanning tree (MST) was constructed to visualize the allelic differences between the isolates. The clusters were created with a threshold of 24 or fewer allelic differences (36).

### Clonal analyses.

BURP algorithm (“based upon repeat patterns”)—as implemented in the SeqSphere + software (Ridom, Germany)—was used to cluster unique spa-types obtained in our study, as we have the sequence of spa-HRM positive controls available. Spa-types shorter than five repeats were excluded from the further analysis due to their limited evolutionary history information.

For each unique spa-type, correlating STs were assigned either by sequencing or from the relevant literature. Spa-types frequently stated in other studies in an ST context were not subjected to sequencing and assigned to the STs from the studies. STs were analyzed by the BURST (“based upon related ST”) approach via the PubMLST website (https://pubmlst.org/organisms/*staphylococcus*-aureus). Clonal complexes (CCs) analysis was carried out at the level of double-loci variants (DLVs). The correlation of isolates’ relatedness based on BURP, BURST, and mini-MLST was studied.

### Data availability.

The raw sequencing data were deposited in the SRA database under the BioProject accession number PRJNA720720.
