# Supplementary material for: Sequencing Independent Molecular Typing of Staphylococcus aureus Isolates: Approach for Infection Control and Clonal Characterization
Source: Microbiol Spectr. 2022 Feb 9;10(1):e01817-21. doi: 10.1128/spectrum.01817-21 (PMC8826877; doi:10.1128/spectrum.01817-21)

Supplementary Table S1

The summary of *S. aureus* data acquired by mini-MLST and spa-HRM. The MLST STs and MLST CCs highlighted in bold indicate the sequencing provided in our laboratory. The non-bold MLST STs and MLST CCs are marked with a symbol referring to the publication from which the data were obtained.

MelT	spa-type	MRSA	MSSA	spa repeat	lenght (bp)	ST	CC
**MelT 224**	t689	1	0	11-19-12-22-25	255	**8**	**8**
	t190	0	1	11-17-34-24-34-22-25	303	**8**	**8**
	t648	0	1	11-21-17-34-24-34-22-25	327	**8**	**8**
	t024	3	254	11-12-21-17-34-24-34-22-25	351	**8**	**8**
	t18941	0	2	11-19-21-17-194-24-34-22-25	351	**8**	**8**
	t1576	1	1	11-12-21-17-34-34-24-34-22-25	375	**8**	**8**
	t1709	1	0	11-19-12-21-17-34-24-34-22-24	375	**4747**	**8**
	t008	15	5	11-19-12-21-17-34-24-34-22-25	375	**8**	**8**
	t211	3	0	11-19-12-12-21-17-34-24-34-22-25	399	**4750**	**8**
	t1767	6	2	11-19-12-21-17-34-24-24-34-22-25	399	**8**	**8**
**MelT 351**	t586	12	0	26-16	183	**225**	**5**
	t151	1	0	26-17-20-17-16	255		5(1)
	t564	1	0	26-17-17-17-16	255	**225**	**5**
	t045	15	0	26-17-20-17-12-17-16	303	**225**	**5**
	t003	146	0	26-17-20-17-12-17-17-16	327	**225**	**5**
	t1623	1	0	07-17-20-17-12-17-17-16	328	**225**	**5**
	t014	18	0	26-17-20-17-12-17-17-17-16	351	**225**	**5**
	t481	1	0	26-17-20-17-12-12-17-17-16	351	**225**	**5**
	t626	1	0	26-17-20-17-12-17-17-16-16	351	**225**	**5**
	t1323	1	0	26-17-20-17-12-17-12-17-17-16	375	**225**	**5**
	t18037	2	0	26-17-20-17-12-17-20-17-12-17-17-17-16	447	**225**	**5**
**MelT 474**	t3698	0	1	08-12-23-02-12-23	280	**6695**	**15**
	t335	0	1	07-23-12-34-34-12-23	303	15(2)	15(2)
	t18007	0	27	26-23-12-34-23-02-12-23	327	**15**	**15**
	t360	0	2	07-23-12-34-12-23-02-12-23	351	15(3)	15(3)
	t2216	0	2	07-23-34-34-12-23-02-12-23	351	15(4)	15(4)
	t346	0	3	07-23-12-34-12-12-23-02-12-23	375	15(3)	15(3)
	t368	0	1	26-23-12-34-12-12-23-02-12-23	375	**15**	**15**
	t085	0	2	07-23-12-34-34-12-23-02-12-23	375	15(3)	15(3)
	t084	5	19	07-23-12-34-34-12-12-23-02-12-23	399	15(3)	15(3)
	t4309	0	1	07-23-12-12-34-34-12-23-02-12-23	399		15(5)
	t5721	0	1	07-34-12-12-23-02-12-23-02-12-23	399	**15**	**15**
	t120	0	1	07-23-12-12-34-34-12-12-23-02-12-23	422	15(6)	15(6)
	t774	0	3	07-23-12-34-34-12-12-12-23-02-12-23	423	15(2)	15(2)
	t1727	0	2	07-23-12-34-34-34-34-12-12-23-02-12-23	447	**582**	**15**
**MelT 404B**	t918	1	0	09-02	183	**45**	**45**
	t026	5	0	08-16-34	207	**45**	**45**
	t635	1	0	08-16-13	207	**45**	**45**
	t1040	0	3	08-16-34-34	231	**45**	**45**
	t728	0	1	08-16-34-16-34	255	45(3)	45(3)

	t18036	0	1	09-34-17-34-34	255	**6289**	**45**
	t3000	0	1	09-02-17-34-16-34	279	**45**	**45**
	t095	0	6	08-16-02-16-34-34	279	**45**	**45**
	t1081	0	3	08-16-02-43-34-17-34	303	**5246**	**45**
	t130	0	2	09-34-13-17-34-16-34	303	**45**	**45**
	t5032	0	1	08-16-02-16-13-17-34-34	327	**278**	**45**
	t065	0	5	09-02-16-34-13-17-34-16-34	351	**45**	**45**
	t1248	0	1	09-02-16-34-13-17-34-16-13	351	6 alleles	**45**
	t737	1	1	08-16-34-34-13-17-34-16-34	351	**45**	**45**
	t18010	0	2	09-02-16-34-34-34-34-16-34	351	**45**	**45**
	t330	0	2	09-02-16-34-34-17-34-16-34	351	**45**	**45**
	t2275	0	1	09-02-16-34-13-17-13-16-34	351	**45**	**45**
	t2623	0	1	08-16-02-16-13-17-13-16-34	351	**45**	**45**
	t1510	1	0	08-16-02-16-34-13-17-34-16-13	375	**45**	**45**
	t116	1	1	08-16-02-16-13-13-17-34-16-34	375	**45**	**45**
	t015	2	3	08-16-02-16-34-13-17-34-16-34	375	**45**	**45**
	t18694	0	2	08-16-34-13-13-17-34-16-13-13	375	**45**	**45**
	t589	0	1	08-16-02-16-34-34-13-17-34-16-34	399	508(2)	45(2)
	t069	0	1	08-16-02-16-34-13-17-34-16-16-34	399	**45**	**45**
	t1231	0	2	08-16-02-16-34-13-17-34-16-34-16-34	423	**45**	**45**
**MelT 349**	t2302	0	1	26-23-17-34	231	**5**	**5**
	t688	1	0	26-23-17-34-17-16	279	**5**	**5**
	t18022	0	1	07-23-17-34-17-20-16	303	**5**	**5**
	t18046	2	0	26-23-22-17-12-17-16	303	**5**	**5**
	t212	0	1	26-17-20-17-12-12-16	303	**5**	**5**
	t010	11	1	26-17-34-17-20-17-12-17-16	351	**5**	**5**
	t105	1	0	26-23-17-34-17-20-17-17-16	351	**5**	**5**
	t1303	0	2	26-17-34-17-20-17-12-12-16	351	**5**	**5**
	t179	2	13	26-23-17-34-17-20-17-12-12-16	375	**5**	**5**
	t242	0	3	26-23-17-13-17-20-17-12-17-16	375	**5**	**5**
	t18041	1	0	135-23-17-34-17-20-17-12-17-16	375	6 alleles	**5**
	t002	4	8	26-23-17-34-17-20-17-12-17-16	375	**5**	**5**
**MelT 251B**	t10060	0	5	07-02-25-34-25	255	**398**	**398**
	t11729	0	3	08-16-34-24-24-25	279	**398**	**398**
	t1451	0	2	08-16-02-25-34-25	279	**398**	**398**
	t011	5	0	08-16-02-25-34-24-25	303	**398**	**398**
	t18045	0	1	08-12-16-02-25-34-25	303	**398**	**398**
	t571	0	6	08-16-02-25-02-25-34-25	327	**398**	**398**
	t034	22	0	08-16-02-25-02-25-34-24-25	351	**398**	**398**
	t17741	1	0	08-16-02-25-02-25-34-24-34-24-25	399	**398**	**398**
**MelT 194**	t1509	0	1	07-23-12-23	231	6 alleles	**7**
	t803	0	7	07-23-02-12-23	255	6 alleles	**7**
	t867	0	2	07-23-17-34-12-23-02-12-23	351		7(7)
	t2932	0	1	07-23-21-17-34-12-23-12-23	351	**7**	**7**
	t091	0	22	07-23-21-17-34-12-23-02-12-23	375	7(4)	7(4)
**MelT 118B**	t338	0	2	15-21-16-02-25-17-24	303		30(8)
	t1347	0	1	08-02-16-02-25-17-24	303	30(9)	30(9)
	t019	0	2	08-16-02-16-02-25-17-24	327	30(4)	30(4)
	t665	2	1	15-12-16-16-02-16-02-25	327		30(10)
	t122	1	3	08-16-02-16-02-25-17-24-24	351	30(11)	30(11)
	t18351	0	2	08-16-784-16-02-25-17-24-24	351	**30**	**30**

	t1641	0	1	15-12-16-02-16-02-25-16-17-24	375	30[(12)]	30[(12)]
	t318	1	0	15-12-16-16-02-16-02-25-17-24	375	30[(4)]	30[(4)]
	t3508	0	1	15-12-16-02-16-02-31-25-17-24-24	399	**30**	**30**
**MelT 423**	t127	13	2	07-23-21-16-34-33-13	303	**1**	**1**
**MelT 357**	t901	0	1	07-23-12-17-20-17-12-12-17	351	**72**	**72**
	t3092	1	0	07-23-12-21-12-17-20-17-12-17	375	72[(2)]	72[(2)]
	t148	0	10	07-23-12-21-12-17-20-17-12-12-17	399	**72**	**72**
	t16914	0	1	07-23-12-21-12-17-20-17-12-12-20-17	423	**72**	**72**
	t1346	0	1	07-23-12-21-12-17-20-17-12-12-12-17	423	72[(2)]	72[(2)]

Number of isolates tested by MLST within a spa type (in case >1 isolates detected within a spa-type): t024 (n = 57), t18941 (n = 1), t1576 (n = 1), t008 (n = 1), t211 (n = 1), t1767 (n = 1), t586 (n = 1), t045 (n = 1), t003 (n = 2), t014 (n = 2), t18037 (n = 1), t18007 (n = 2), t1727 (n = 1), t026 (n = 1), t1040 (n = 1), t095 (n = 1), t1081 (n = 2), t130 (n = 1), t065 (n = 1), t737 (n = 1), t18010 (n = 1), t330 (n = 1), t116 (n = 1), t015 (n = 1), t18694 (n = 1), t1231 (n = 1), t18046 (n = 1), t010 (n = 2), t1303 (n = 1), t179 (n = 2), t242, t002 (n = 1), t10060 (n = 1), t11729 (n = 1), t1451 (n = 1), t011 (n = 1), t571 (n = 1), t034 (n = 1), t18351 (n = 1), t127 (n = 1), t148 (n = 2).

REFERENCES

1. Garbacz K, Piechowicz L, Podkowik M, Mroczkowska A, Empel J, Bania J. 2018. Emergence and spread of worldwide *Staphylococcus aureus* clones among cystic fibrosis patients. Infect Drug Resist 11:247-255.

2. Conceição T, Coelho C, Silva IS, de Lencastre H, Aires-de-Sousa M. 2015. *Staphylococcus aureus* in former Portuguese colonies from Africa and the Far East: missing data to help fill the world map. Clin Microbiol Infect 21:842.

3. Skråmm I, Moen AE, Bukholm G. 2011. Nasal carriage of *Staphylococcus aureus*: frequency and molecular diversity in a randomly sampled Norwegian community population. APMIS 119:522-528.

4. Lepuschitz S. 2015. Subtyping of livestock-associated methicillin-resistant *Staphylococcus aureus* CC398 isolates by next generation sequencing [master's thesis]. Vienna (AU): University of Vienna.

5. Krupa P, Bystroń J, Podkowik M, Empel J, Mroczkowska A, Bania J. 2015. Population structure and oxacillin resistance of *Staphylococcus aureus* from pigs and pork meat in south-west of Poland. Biomed Res Int 2015:141475.

6. Skråmm I, Moen AE, Alm-Kristiansen K, Bukholm G. 2007. Nasal carriage of *Staphylococcus aureus*: which sequence types do orthopedic surgical healthcare workers carry? Infect Control Hosp Epidemiol 28:737-739.

7. Donker GA, Deurenberg RH, Driessen C, Sebastian S, Nys S, Stobberingh EE. 2009. The population structure of *Staphylococcus aureus* among general practice patients from The Netherlands. Clin Microbiol Infect 15:137-143.

8. Balma-Mena A, Lara-Corrales I, Zeller J, Richardson S, McGavin MJ, Weinstein M, Pope E 2011. Colonization with community-acquired methicillin-resistant *Staphylococcus aureus* in children with atopic dermatitis: a cross-sectional study. Int J Dermatol 50:682-688.

9. Li QT, Zhu YZ, Dong K, Liu C, Zhou YH, Ni YX, Guo XK. 2011. A novel sequence-based coa genotyping method to discriminate nosocomial methicillin-resistant *Staphylococcus aureus* isolates. Ir J Med Sci 180:463-468.

10. Pardos de la Gandara M, Raygoza Garay JA, Mwangi M, Tobin JN, Tsang A, Khalida C, D'Orazio B, Kost RG, Leinberger-Jabari A, Coffran C, Evering TH, Coller BS, Balachandra S, Urban T, Parola C, Salvato S, Jenks N, Wu D, Burgess R, Chung M, de Lencastre H, Tomasz A. 2015. Molecular types of methicillin-resistant *Staphylococcus aureus* and methicillin-sensitive *S. aureus* strains causing skin and soft tissue infections and nasal colonization, identified in community health centers in New York City. J Clin Microbiol 53:2648-2658.

11. Golding GR, Levett PN, McDonald RR, Irvine J, Quinn B, Nsungu M, Woods S, Khan M, Ofner-Agostini M, Mulvey MR. 2011. High rates of *Staphylococcus aureus* USA400 infection, Northern Canada. Emerg Infect Dis 17:722-725.

12. Lozano C, Gómez-Sanz E, Benito D, Aspiroz C, Zarazaga M, Torres C. 2011. *Staphylococcus aureus* nasal carriage, virulence traits, antibiotic resistance mechanisms, and genetic lineages in healthy humans in Spain, with detection of CC398 and CC97 strains. Int J Med Microbiol 301:500-505.

Supplementary Figure S1

Representative melting curves of mini-MLST typing for *S. aureus*.

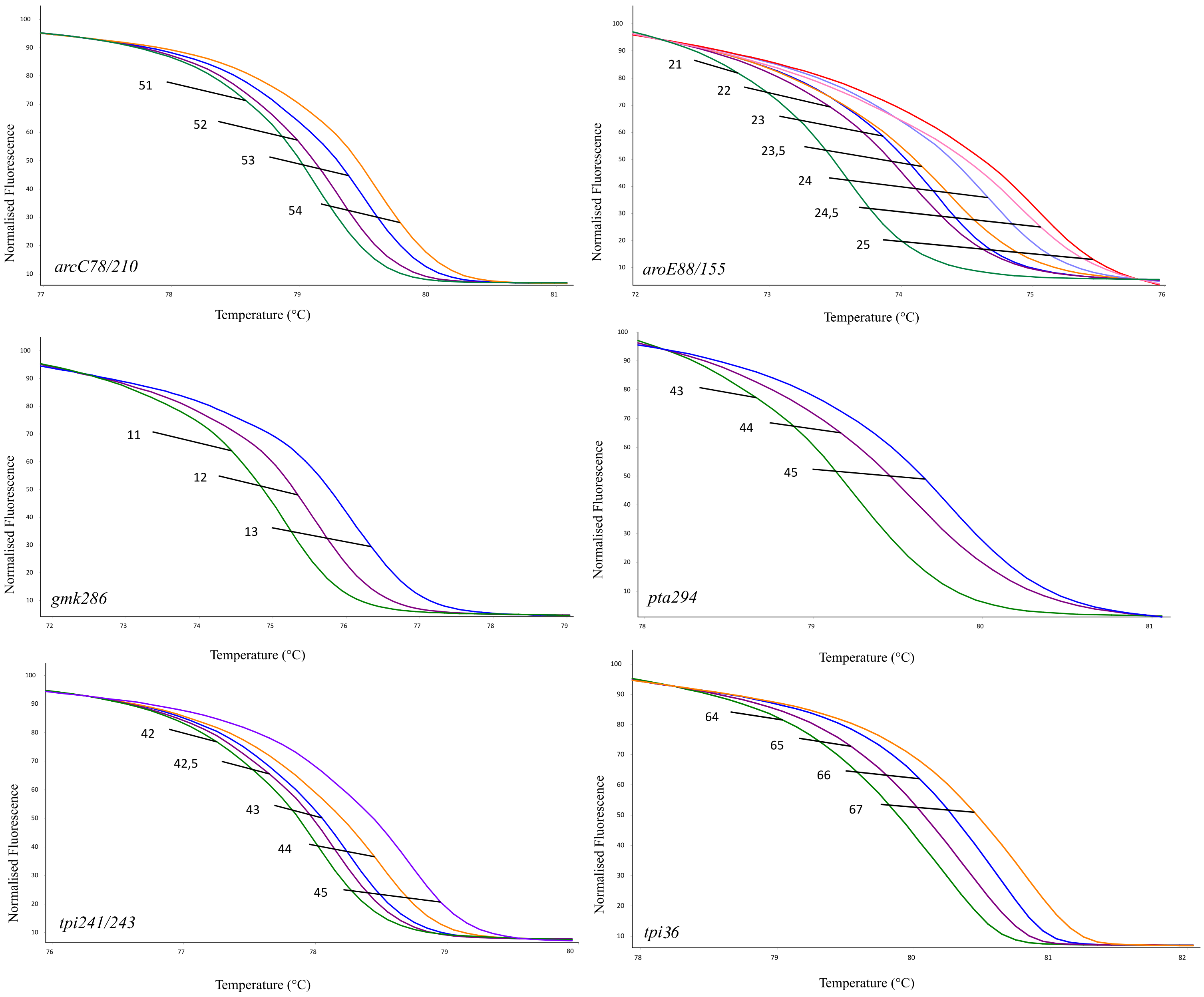

Supplementary Figure S2

Mini-MLST demonstration of 38 samples for *gmk286* locus by both normalised melting curves (A) and difference curves (B).

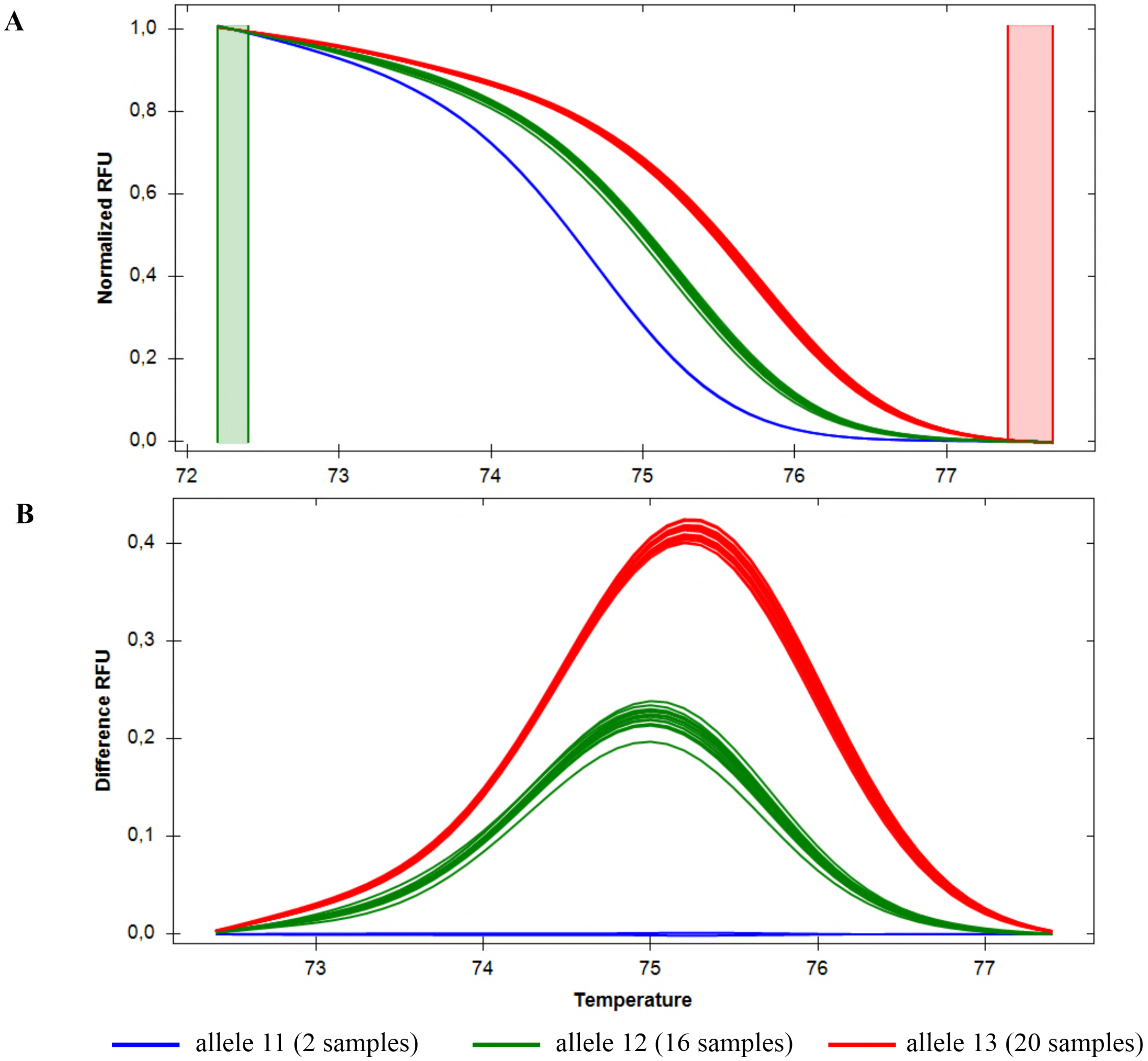

Supplementary Figure S3

Representative melting curves of spa-HRM typing performed for the prevalent MelTs observed in our study (except MelT404B).

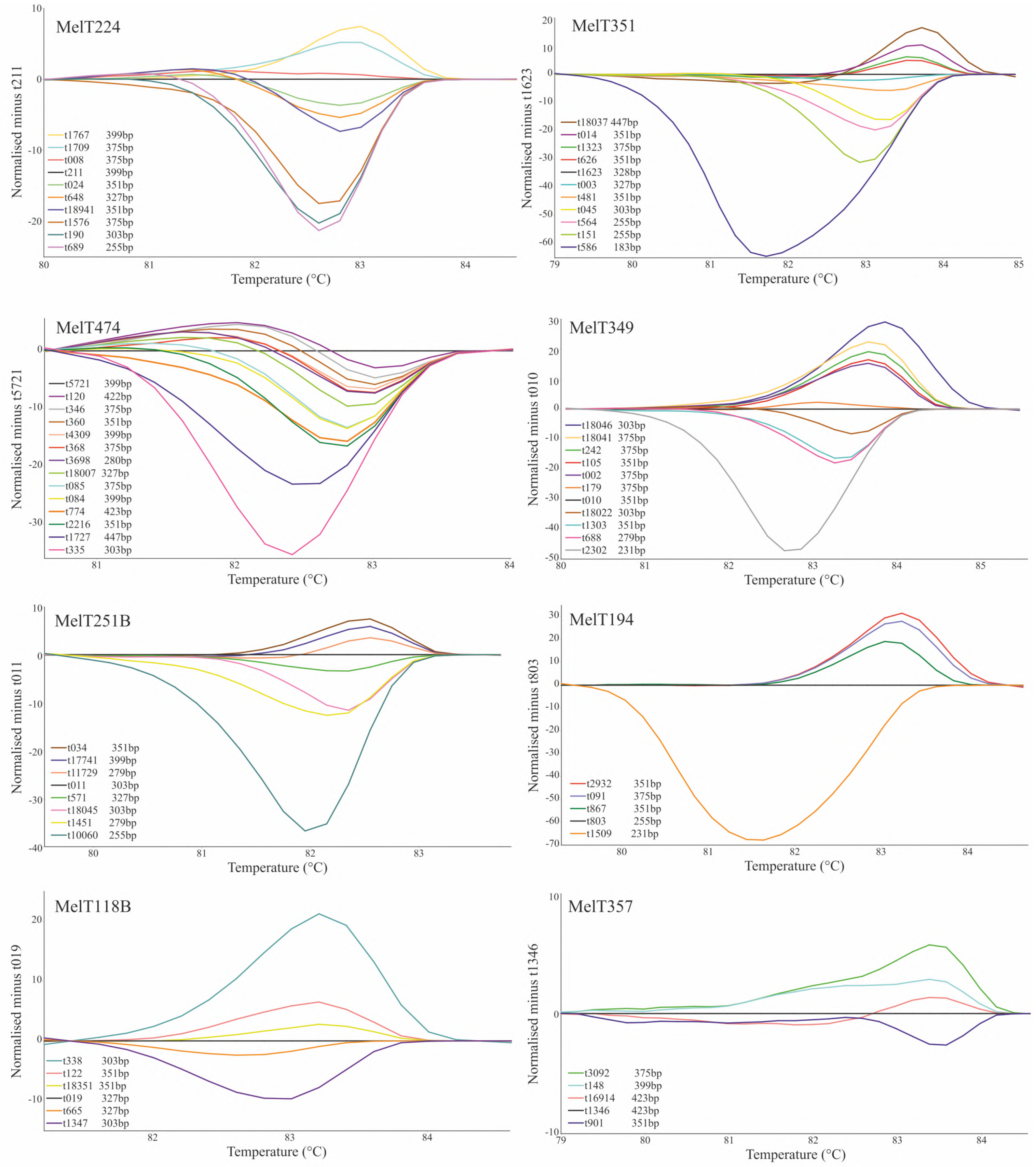

Supplementary Figure S4

Representative melting curves of spa-HRM typing performed for the MelT404B.

Pre-segregation was done based on the lenght of the PCR product.

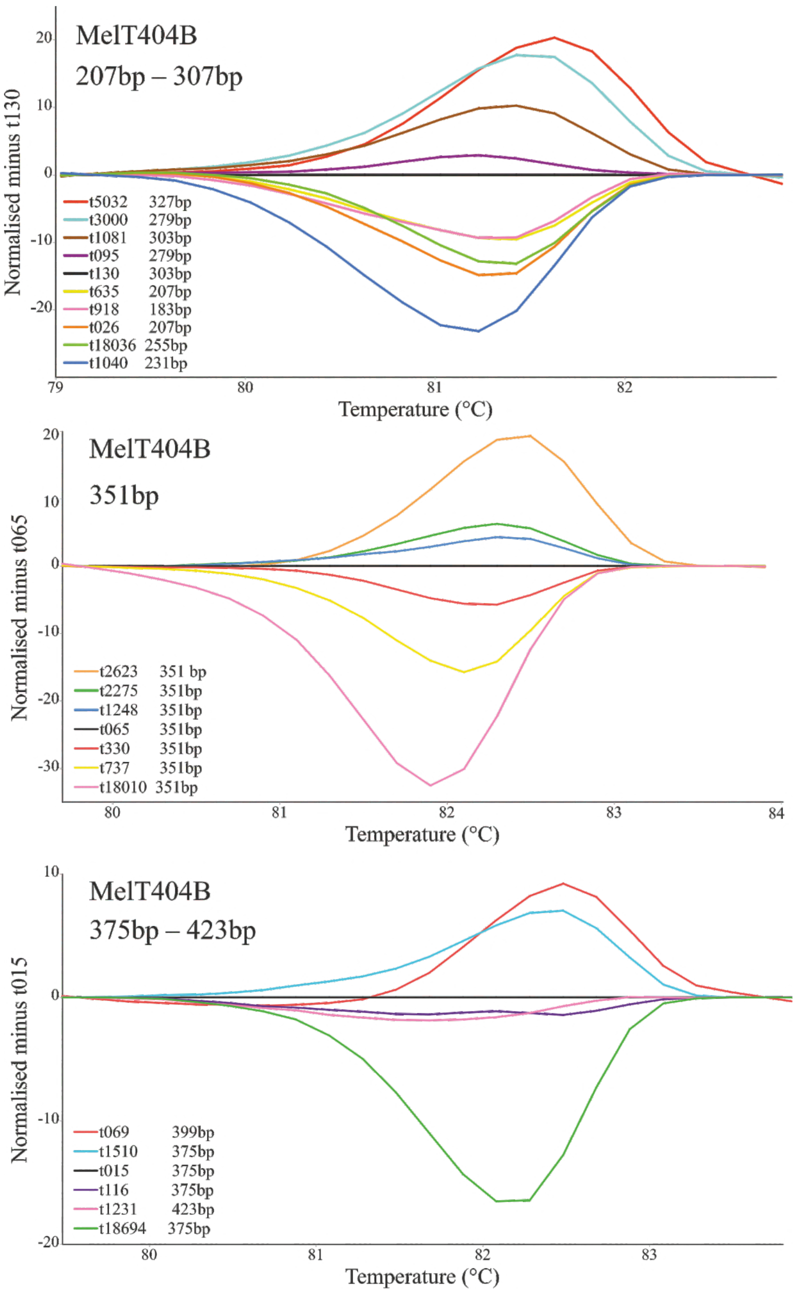

Supplement: SUPPLEMENTAL FILE 1 — Supplemental material. Download SPECTRUM01817-21_Supp_1_seq8.pdf, PDF file, 5.6 MB [file spectrum01817-21_supp_1_seq8.pdf]
